# The Role of Vegetable Genetic Resources in Nutrition Security and Vegetable Breeding

**DOI:** 10.3390/plants9060736

**Published:** 2020-06-11

**Authors:** Andreas W. Ebert

**Affiliations:** World Vegetable Center, 60 Yi-Min Liao, Shanhua, Tainan 74151, Taiwan; ebert.andreas6@gmail.com

**Keywords:** malnutrition, food security, vegetables, genetic resources, ex situ conservation, home gardens, community seedbanks, variety introduction, vegetable breeding

## Abstract

Malnutrition, comprising undernutrition, micronutrient deficiency, and overnutrition, is more widespread than hunger per se and affects most nations around the globe. The diversity and the quality of food produced and consumed are decisive factors when addressing the triple burden of malnutrition. In this context, fruit, vegetables, and nuts are increasingly moving into the focus of the nutrition community. Agricultural policies and investments in agriculture are predominantly focused on staple food production, neglecting the economic and nutritional potential of fruit and vegetables. While global vegetables are well represented in genebanks around the globe, this is much less the case for traditional vegetables. Collecting efforts in hotspots of vegetable diversity in Africa and Asia are required to conserve this germplasm before it is being replaced by modern varieties. Home gardens, community seedbanks, and variety introduction through vegetable seed kits are ways how genebanks can link with the farming community to strengthen the informal seed sector. This in turn may result in more diverse production systems and increased consumption of fruit and vegetables. In the formal seed sector, vegetable breeders need access to a wide diversity of genetic resources, predominantly farmers’ varieties, landraces, and crop wild relatives. Genomics-assisted breeding is increasingly facilitating the introgression of favorable genes and quantitative trait loci (QTLs) with complex inheritance patterns from wild species into cultigens. This will lead to wider use of crop wild relatives in the development of resilient cultivars.

## 1. Introduction

The Green Revolution had positive impacts on poverty reduction and lower food prices [1]. These effects were mostly driven by crop germplasm improvement programs of the CGIAR centers, resulting in impressive yield increases per hectare. The CGIAR is a global agricultural research partnership for a food secure future that was founded in the 1970s. The new, improved varieties were taken up by national agricultural agencies for adoption and broad-scale dissemination among the farming communities. From 1960 to 2000, yields across all developing countries increased 208% for wheat, 109% for rice, 157% for maize, 78% for potatoes, and 36% for cassava [2]. Estimates of the impact of crop germplasm improvement alone indicate average productivity gains, across all regions of the world, of 1.0% per annum for wheat, 0.8% for rice, 0.7% for maize, 0.6% for millets, and 0.5% for sorghum [3].

The historic success of the Green Revolution in terms of yield gains, together with lower food prices, ensured adequate quantities of staple cereal grain, thereby drastically reducing the problem of famine. However, after years of steady decline, the trend in world hunger reverted in 2015 and is now slowly increasing. About 820 million people or almost 11% of the global population suffered from chronic undernourishment in 2018 [4]. This underscores the immense challenge of achieving the Zero Hunger target of the 2030 Agenda for Sustainable Development adopted by the United Nations (UN) in 2015, which comprises 17 Sustainable Development Goals (SDGs). As stated by the United Nations Development Program [5], the SDGs were adopted as a “call for global action to end poverty, protect the planet, and ensure that all people enjoy peace and prosperity by 2030”. SDG 2—Zero Hunger calls for the eradication of hunger and all forms of malnutrition, with targets for doubling agricultural productivity and incomes of small-scale food producers (SDG 2.3), ensuring sustainable food systems (SDG 2.4), and maintaining genetic diversity (SDG 2.5).

Global demand for agricultural crops is predicted to increase until 2050 due to population growth, greater per capita purchasing power which translates into higher meat and dairy consumption, and biofuel use [6,7,8,9]. Analyses undertaken by Tilmann et al. [8] revealed that per capita demand for crop calories or protein is, since 1960, similarly dependent on per capita real income within and among seven economic groups. Those groups ranged from highest (group A) to lowest (group G) national average per capita inflation-adjusted gross domestic product (GDP). By 2050, global crop production should be 60% to 110% higher than that of 2005/2007 to feed the expected world population of more than nine billion [8,9,10]. Depenbusch and Klasen [11] estimated a 61% increase in calorie requirements between 2010 and 2100 if weight per age–sex group would remain stable. Considering the current trend toward increased human height and weight at a given height, expressed in terms of the average body mass index (BMI), this might add another 18.7 percentage points to the projected 61% increase in future national and global caloric requirements. Thus, an additional significant increase in food production would be required to satisfy demand.

Malnutrition, comprising undernutrition, micronutrient deficiency, and overnutrition, is more widespread than hunger per se and affects most nations around the globe. According to the 2018 Global Nutrition Report [12], children under five years of age face multiple burdens: stunting (low height for a child’s weight) affects 150.8 million, wasting (low weight for a child’s height) affects 50.5 million, and overweight (BMI at or above the 85th percentile and below the 95th percentile for children and teens of the same age and sex [13]) affects 38.3 million. Each year, 20 million babies are born of low birth weight (less than 2500 g) and are 20 times more likely to die in infancy than heavier babies. In 2016, 131 million children 5–9 years old, 207 million adolescents, and two billion adults were overweight [4]. About one-third of overweight adolescents and adults, and 44% of overweight children aged 5–9 were obese. Thus, overweight and obesity are at an alarming rate globally. Obesity is defined as a BMI at or above the 95th percentile for children and teens of the same age and sex [13]. In adults, BMI values of 30.0 or higher fall within the obese range.

Malnutrition is the single most important risk factor for disease. Diet-related diseases such as diabetes, cardiovascular disease, hypertension, stroke, cancer, and obesity are escalating at a global level. In 2016, an estimated 40.5 million (71%) of the 56.9 million deaths worldwide were caused by non-communicable diseases (NCDs) [14]. Approximately 32.2 million NCD deaths (80%) were attributable to cancers, cardiovascular diseases, chronic respiratory diseases, and diabetes, whereas the remaining 8.3 million (20%) were caused by other NCDs. These figures illustrate the seriousness of diet-related diseases for the healthcare sector. NCDs now pose a greater risk to morbidity and mortality than unsafe sex, alcohol, and drug and tobacco use combined [15].

To address SDG 2, it becomes obvious that there is a clear need to reorient agricultural production from a mere increase in food quantity toward delivery of more diverse and more nutritious food produced in a sustainable manner [15,16]. As incomes rise and food consumption patterns change, overnutrition from imbalanced diets also becomes a matter for concern, both in developed and in developing countries. The diversity and quality of food produced and consumed is a decisive factor when addressing the triple burden of malnutrition, i.e., undernutrition, micronutrient deficiency, and overnutrition.

Although fruit and vegetables are usually mentioned jointly when addressing malnutrition, this paper focuses mainly on the role of vegetables for nutrition security, their global production, and the current ex situ conservation of both global and traditional vegetables. The need for additional conservation efforts in hotspots of vegetable diversity to safeguard valuable germplasm for current and future breeding efforts is addressed. Ways in which genebanks can link with the farming communities to strengthen the informal seed sector and to introduce genetic diversity at the farm and household level are briefly described. In the formal seed sector, vegetable breeders need access to a wide diversity of genetic resources, predominantly farmers’ varieties, landraces, and crop wild relatives (CWR). The use of conventional and modern breeding tools to make efficient use of CWR for the development of resilient cultivars is briefly discussed.

## 2. The Importance of Vegetables for Nutrition Security

The rise of modern agriculture is generally considered to have led to a decline in the number of plant species upon which humans depend for food [17,18]. This decline particularly affected the wild, semidomesticated, and cultivated traditional vegetables and fruits, spices, and other food plants that supplemented staple crops with micronutrients, essential for a healthy diet. Those crops/species also strengthened food security historically during failures of the main crops [19]. More recent analyses of food consumption trends, regionally and globally, revealed increases in the diversity of plants contributing to diets locally in developing countries [20]. These changes are driven by rising income, urbanization, trade liberalization, transnational food corporations, and food industry marketing and retailing through supermarkets. Consumers are now offered more diversity and convenience but at the same time cheaper, less healthy, and highly processed food with high content of fats and sugars has been made more easily accessible and affordable and is, therefore, in high demand [21]. These developments led to a Westernization transition of local diets with a preference for energy-dense foods, such as animal products, plant oils, and sugars over cereals, pulses, and vegetables, with a general preference for more global crop plants over locally produced traditional crops [22,23].

A recent study examined changes in the richness, abundance, and composition of crop commodities in the food supplies of 142 countries (comprising 98% of the global population from 1961 to 2009 [24]. During this 50-year period, national food supplies expanded quantitatively in terms of food calories, protein, fat, and weight, and significant quantities of food consumed were derived from energy-dense food sources, known to contribute to malnutrition. Because of these global food consumption trends, national food supplies became more similar in composition globally, thanks to a constant flow of a range of truly global cereal and oil crops, while the supply of local traditional crops declined. The growing reliance on the supply of those global food crops is leading to stronger interdependence among countries in their food supplies, underlying genetic resources, and nutritional priorities.

It is increasingly recognized that the global food system must shift its focus from food quantity toward dietary quality and health and environmental outcomes [15,16]. Fruit, vegetables, and nuts are increasingly entering into the focus of the nutrition community for their potential in combating the triple burden of malnutrition [25]. Traditional, underutilized crops, especially those which are locally available and culturally acceptable, are ideally placed to play a much greater role in contributing to improved nutrition and health [26], in line with the strategy proposed by the British Royal Society: “The preferred strategy to eliminate hidden hunger will always involve strategies to increase the diversity of diet with increased access to fruit and vegetables” [27]. The World Health Organization (WHO) recommends a population-wide daily intake of 400 g of edible fruit and vegetables for the prevention of NCDs, as well as for the prevention and alleviation of several micronutrient deficiencies [28]. This WHO recommendation translates to roughly five portions per day. It should be noted that potatoes, sweet potatoes, cassava, and other starchy roots are not considered as fruits or vegetables.

People able to enjoy more diverse diets, in general, also have better nutrition and health. A recent study analyzing data of a health survey in Great Britain revealed that there is a robust inverse association between fruit and vegetable consumption and mortality [29]. A study of Helen Keller International’s integrated household food production program targeting women in Burkina Faso found significant improvements in several child nutrition indicators, including reductions in anemia, wasting, and diarrhea among young children [30]. The underlying agriculture production activities of this integrated program included input distribution (seeds, saplings, chicks, and gardening tools) and agricultural training. Household food production activities focused primarily on micronutrient-rich vegetables and fruits, eggs, and chicks.

Increasing production and consumption of fruit and vegetables is an obvious pathway to improve dietary diversity and enhance nutrition security, especially in the case of diets that are dominated by high-energy foods with low levels of micronutrients [29]. Such a move, coupled with a transition to diets higher in plant-based protein, will also help protect valuable habitats such as the Amazon rainforest and help meet the SDGs [31]. However, several studies suggested that current and projected fruit and vegetable production levels will fail to meet healthy consumption levels [31,32]. Following age-specific recommendations, only 40 countries representing 36% of the global population had adequate availability of fruit and vegetables by 2015 [33]. In many food-insecure countries in sub-Saharan Africa and South Asia, average fruit and vegetable consumption is well below WHO-recommended levels, with 10 countries not even meeting 30% of the recommended intake levels [34].

Similarly, projections to 2050 indicate that between 0.8 and 1.9 billion people living in countries in sub-Saharan Africa might need to contend with average fruit and vegetable availability below 400 g/person per day [33]. This estimate does not yet take food waste into consideration. Under a high-food-waste scenario, these authors predict that 139 countries representing 5.6 billion people will not be able to provide enough fruit and vegetables to the population by 2050—an increase of 1.5 billion people compared with a no-waste scenario. Reducing waste in vegetable and fruit value chains may also reduce the negative impact of their production on the environment and could keep consumer prices more accessible [35].

### 2.1. The Commodity Group “Vegetables”

Vegetables comprise a wide range of genera and species and are an important component of a healthy diet. They ensure nutrition security through the provision of vitamins, antioxidants, minerals, fiber, amino acids, and other health-promoting compounds [36], while enhancing diversity, flavor, and taste of many otherwise bland staple dishes. Due to their wide range of uses, it is difficult to assign crops to the commodity group “vegetables”. Some legume crops, mainly grown for their dry seeds, are important vegetable crops when harvested and consumed at the immature stage as seeds, green pods, and/or leaves, as is common in Asia and Africa. This applies, for example, to crops like vegetable soybean (*Glycine*), mungbean (*Vigna radiata*), Azuki beans (*Vigna angularis*), cowpea (*Vigna unguiculata*), yard-long bean (*Vigna unguiculata* subsp. *sesquipedalis*), black gram (*Vigna mungo*), common bean (*Phaseolus*), winged bean (*Psophocarpus*), and garden pea (*Pisum*). Although the starchy root of cassava (*Manihot esculenta*) constitutes the primary use of this crop, cassava leaves are a common leafy vegetable in many African countries; hence, this crop also falls partially under vegetables.

The most dominant vegetables, also called global vegetables as they are grown in many countries around the globe, are tomatoes, cucurbits (pumpkins, squashes, cucumbers, and gherkins), alliums (onion, garlic, shallot), and chilies (sweet and hot pepper; *Capsicum* spp.). Other major vegetable crops based on farmgate value of global production, but not always truly global vegetables are spinach, brassicas (cabbages, broccoli, rape), vegetable legumes, eggplants, lettuce and chicory, carrots and turnips, and asparagus [37]. Production statistics usually do not list indigenous or traditional vegetables as these are often produced in home or family gardens or collected from the wild for family consumption, and they are, in general, only offered in local markets. The term “indigenous vegetables” primarily refers to plants grown in their centers of origin or diversity [38], but also encompasses plant species introduced from other geographical areas that adapted well, naturalized, and evolved in the new environment [39]. Indigenous vegetables are often more nutrient-dense than global vegetables [40], require low levels of external inputs, and cope well with abiotic and biotic stresses if grown on a small scale and in mixed cropping systems as is the case in their centers of origin. However, data on nutritional profiles of indigenous vegetables in raw and cooked forms are scarce. Among the traditional vegetables with high potential to be mainstreamed into urban markets and consumer diets are leafy amaranth (*Amaranthus* spp.), African eggplant (*Solanum macrocarpon*), jute mallow (*Corchorus olitorius*), roselle (*Hibiscus sabdariffa*), spider plant (*Cleome gynandra*), Ethiopian mustard (*Brassica carinata*), okra (*Abelmoschus esculentus*), and vegetable cowpea (*Vigna unguiculata*) [41].

### 2.2. Global Vegetable Production

**Figure 1:**
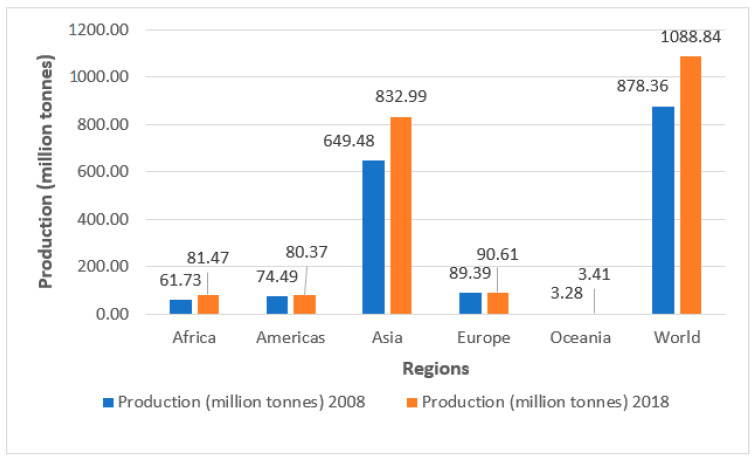
Comparison of global production of primary vegetables in 2008 and 2018, by major regions (Source: Statistics Division of FAO). Available online: http://www.fao.org/faostat/en/#data/QC/ (accessed on 14 March 2020).

**Figure 2:**
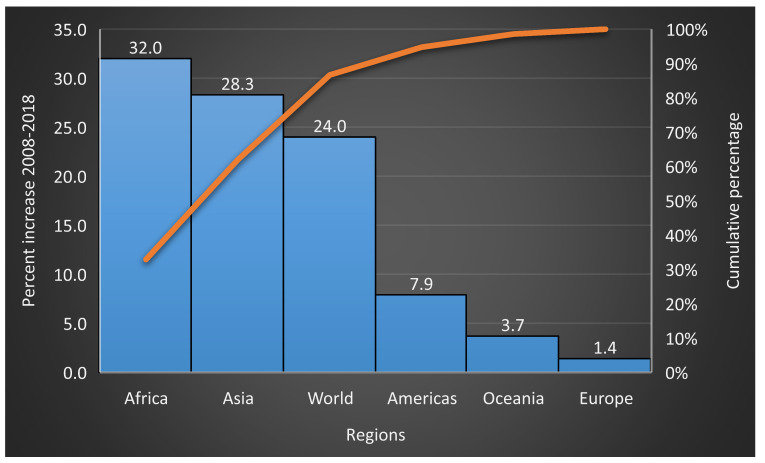
Percentage production increase of primary vegetables from 2008 to 2018 (calculated from production figures retrieved from Statistics Division of FAO). Available online: http://www.fao.org/faostat/en/#data/QC/ (accessed on 14 March 2020).

The statistics of the Food and Agriculture Organization of the United Nations (FAO) cover 25 primary vegetable products (http://www.fao.org/faostat/en/#data/QC). Global primary vegetable production reached 1.09 billion tons in 2018, about 37% of global cereal production (2.96 billion tons) [42]. Asia is by far the largest producer of primary vegetables, responsible for three-quarters of global production (Figure 1). During the past 10 years (2008–2018), there was a 24% increase in global commercial vegetable production, mainly attributable to a significant production increase in Africa (32%) and Asia (28.3%) (Figure 2). The estimated farmgate value of annual global vegetable production reached 543 billion United States dollars (US$) in 2012–2013, about 65% of all food cereals combined, estimated at 837 billion US$ [37].

### 2.3. Research and Development Efforts Focusing on Vegetables

Although the farmgate value of annual global vegetable production is impressive when compared to global cereal production, the potential of vegetables to generate positive economic and nutritional impacts is still limited due to a relatively low level of support provided by national governments and development agencies to public sector vegetable research and development (R&D) [43]. Agricultural policies and investments in agriculture by the public and private sectors are still mainly focused on staple food production (cereals, roots, and tubers) that provide the primary source of calories, especially for low-income consumers. Fruit and vegetables, rich in micronutrients and vitamins, are not given appropriate support [44,45]. About 45% of private sector agricultural research and development investments into biotechnology and the crop seed industry are dedicated to a single crop—maize—and this investment is mainly taking place in industrialized countries [46].

With a few exceptions, private and public sector investments in vegetable research are mainly focusing on the development of hybrid cultivars of predominantly global vegetables, such as tomatoes, chilies, lettuce, cucumbers, and onions, while indigenous or traditional vegetables are being neglected. Data for 70 countries compiled by Schreinemachers et al. [37] clearly indicate the underinvestment in vegetable research and development compared to cereals. While there are 4–5 publicly funded cereal researchers per one million inhabitants in all country groups, low- and lower–middle-income countries, on average, have only one researcher working on fruit or vegetables [38]. When research investment in fruit and vegetables is placed in relation to production value of the different commodity groups, investment in fruit and vegetables is about the same for all country groups. However, higher-income countries allocate much higher research investment resources per dollar of cereal output than per dollar of vegetable output.

Investments in fruit and vegetable production should primarily target regions where projected supply is insufficient, such as sub-Saharan Africa, parts of Asia, and the Pacific. As fruit and vegetable production is mostly in the hands of small-scale producers in these regions, investments in this sector would have substantial potential to increase incomes and food security in these regions, particularly in Africa [33], thus contributing to the attainment of SDG 1—no poverty and SDG 2—zero hunger.

More research is also needed with respect to how nutrient profiles of global and traditional vegetables change in relation to cultivar choice, growing conditions, processing, and cooking methods. In addition, it is essential to study the extent of nutrient uptake and absorption by the human body. A study of the factors determining micronutrient bioaccessibility in leafy vegetables revealed that the pectin content of the leaves impaired carotenoid bioaccessibility [47]. Leafy vegetables rich in condensed tannins, such as drumstick tree (*Moringa oleifera*), had exceptionally low content of pectin and were characterized by high micronutrient bioaccessibility. Therefore, selection and development of cultivars with high micronutrient and low pectin content is a good approach to improve absorption of micronutrients by the human gut.

Fruit and vegetables are also quite perishable, with some estimates suggesting that these commodities contribute more than 40% of total food losses and waste [48]. Investments in R&D of vegetables and fruit value chains, focusing on new and improved processing, storage, and distribution technologies, could lead to a significant reduction of food losses and waste. Moreover, value addition through the development of drying technologies and novel extraction methods transforming fruit and vegetable waste (such as pomace, peels, and seeds) into new dehydrated and nutraceutical by-products could lead to a further reduction of food waste [49].

In general, it is important that the research and development community not only focuses on the farm segment of the food system but increasingly widens the understanding and supports the transformation of the entire system. Research strategies must include vegetable processing, the logistics of storage and transport to urban markets, and wholesale of inputs and outputs in the food system [21]. R&D investment efforts in these off-farm segments which account for 40–70% of value added and costs of food merit increased attention and could lead to lower marketing margins in value chains, thus improving efficiency.

As low consumption is also a problem where fruit and vegetable availability is not a constraint, it is important to address necessary changes in consumer behavior, apart from increasing production and availability of such crops. The provision of quantified food-based dietary guidelines is indicated to give the population some orientation, and such guidelines are missing in many countries, especially in Africa [50]. Apart from behavior change communication aimed at encouraging people to adopt healthier food choices that include fruit and vegetables, other key approaches are vegetable home gardens (see below) and school gardens. The latter simultaneously strengthen demand and supply and create awareness of the importance of the inclusion of fruit and vegetables in household and school meals to improve nutrition and health [37].

### 2.4. Diversifying Production Systems and the Role of Home and Household Gardens for Nutrition Security

The growing demand for fruit and vegetables can be met through diversification of staple crop systems by including fruit and vegetable crops or through intensification of specialized fruit and vegetable systems, especially in peri-urban zones [51]. Integrating small-scale farmers with formal and informal market outlets was shown to strongly encourage those farmers to adopt diversified vegetable production and other sustainable intensification practices in Kenya, as these result in economic benefits to farmers [52].

In view of global climate change, diversifying agricultural and horticultural production will help increase resilience of farming systems for both biotic and abiotic stresses. By the end of the 21st century, crop calorific yields in single cropping systems in sub-Saharan Africa are predicted to reach only 40–55% of the crop calorific yields obtained in sequential cropping systems [53]. Multispecies cropping systems constitute practical applications of ecological principles based on biodiversity, plant interactions, and other natural regulation mechanisms [54]. Such systems offer potential advantages in productivity, yield stability, ecological sustainability, and resilience to disruptions caused by climate change and other natural events but are sometimes considered harder to manage effectively than monocultures. A wider use of neglected and undervalued fruit and vegetable crops and semi-domesticated species, either intercropped with main staples in cereal-based systems or as stand-alone crops, would provide multiple options to build temporal and spatial heterogeneity into uniform cropping systems. Such an approach will enhance resilience to biotic and abiotic stress factors which are exacerbated by global climate change and will ultimately lead to a more sustainable supply chain of diverse and nutritious food [55]. Pilot studies conducted in Kenya and Vietnam revealed that diversification of smallholder farms with underutilized and traditional vegetable crops such as African nightshade (*Solanum americanum*), cowpea (*Vigna unguiculata*) crotalaria (*Crotalaria brevidens*), French beans (*Phaseolus vulgaris*), groundnuts (*Arachis hypogea*), kale (*Brassica oleracea* var. *acephala*), pumpkin (*Cucurbita maxima*), purple amaranth (*Amaranthus blitum*), spider plant (*Cleome gynandra*), mustard greens (*Brassica juncea*), orange-fleshed sweet potato (*Ipomoea batatas*), and water spinach (*Ipomoea aquatica*) could cover vitamin A requirements of 10–31 extra people per hectare and enhance income by 25% to 185% [56]. However, a trade-off of these diversification interventions was reduced leisure time.

Apart from such specialized, intensive production systems, home or household garden interventions have a proven effect on nutritional outcomes. Widely practiced forms of gardening consist of the mixed cropping of different species of fruits, vegetables, herbs, spices, and other useful plants as a supplementary source of food and income, and such gardens are common in Asia [57]. A well-planned and well-managed garden is expected to supply the household with a diverse range and year-round harvest of fruit and vegetables for household consumption with possible surplus sold on local markets. Such interventions potentially contribute to eight of the 17 Sustainable Development Goals [58]. The key benefits of home gardening were summarized by Landon-Lane [59] as follows:

- Improved food security,
- Increased availability of food and better nutrition through food diversity,
- Income and enhanced rural employment through additional or off-season production,
- Decreased risk through diversification,
- Environmental benefits from recycling water and waste nutrients, controlling shade, dust, and erosion, and maintaining or increasing local biodiversity.

Home gardens are typically targeted at women as they are, in general, in control of meal choice and preparation, and this may lead to women’s empowerment as shown in Bangladesh [60]. Experiments with home gardens in Hyderabad, southern India, including about two dozen vegetable species, showed that a small area of 6 m × 6 m can provide much of the vitamin A and C requirement for a family of four during the entire year [34]. Apart from the provision of essential vitamins, many of the vegetable crops included in home garden kits are known to be naturally nutrient-dense [40,61,62].

## 3. Vegetable Genetic Resources Conservation and Linkages with the Farming Community

As genetic erosion continues in situ and on-farm due to expansion of human settlements, climate change, and replacement of landraces by high-yielding hybrid cultivars, additional collecting and conservation efforts are mandatory with a major focus on crop wild relatives and poorly represented landraces of major and minor vegetable groups. Special attention is needed to conserve the genetic diversity of indigenous and underutilized vegetable crops which, in general, are poorly conserved.

### 3.1. Ex Situ Conservation of Vegetable Genetic Resources and Collecting Needs

The Second Report on the State of the World’s Plant Genetic Resources for Food and Agriculture (SoWPGR-2) indicates that about 7.4 million accessions are currently maintained ex situ, globally, 1.4 million more than were reported in the first SoW report [63]. Information in the World Information and Early Warning System (WIEWS) on the Plant Genetic Resources for Food and Agriculture (PGRFA) database indicates that about 45% of all the accessions in the world’s genebanks are cereals, followed by food legumes with 15% of global holdings [63]. About one million accessions of crops used fully or partially as vegetables are conserved ex situ [64]. In a narrow sense of crops exclusively used as vegetables, about 518,000 accessions of vegetables representing 7% of the globally held 7.4 million accessions of PGRFA are maintained ex situ [63]. Among vegetable commodities, tomatoes (84,289 accessions), chilies (73,572), brassicas (25,566), cucurbits, excluding melons and cantaloupe (39,583), alliums (29,898), okra (22,428), and eggplant (21,616) are well represented in ex situ collections at the global level [64].

The World Vegetable Center (WorldVeg) with its headquarters in Taiwan holds about 63,500 accessions of vegetable germplasm comprising 170 genera and 456 species from 158 countries of origin [41], including some of the world’s largest vegetable collections held by a single institution, such as chilies, tomato, and eggplant, as well as about 12,000 accessions of indigenous vegetables [40] (https://avrdc.org/our-work/managing-germplasm/). The WorldVeg germplasm collection can either be searched in its own database AVGRIS (http://seed.worldveg.org/search/passport) containing 71,889 passport records, or in the global database Genesys (https://www.genesys-pgr.org/) with 59,954 accession records.

WorldVeg works with Kew Royal Botanic Gardens, United Kingdom (UK) and partners in the Global Crop Wild Relative project to rescue the diversity of eggplant wild relatives from Africa. Through the project, WorldVeg obtained the seeds of 217 accessions of 18 species of eggplant wild relatives [41]. These accessions are currently being multiplied and characterized by WorldVeg genebank staff. Wild relatives of cucurbits, vegetable cowpea, and okra are other priority species in need to be collected in Africa and Asia through partnerships with local players to safeguard landraces and wild relatives of vegetable crops in both continents.

WorldVeg collaborates with National Plant Genetic Resources Centers in Eastern and Southern Africa to identify hotspots of vegetable diversity and to upload vegetable biodiversity data to public databases. In total, 126 high-potential traditional African vegetables relevant for people’s diets in different regions of Africa were identified in collaboration with the World Agroforestry Center and the Inland Norway University of Applied Sciences [41]. Hotspots of vegetable diversity were identified in the coastal regions of West Africa, in Cameroon, South Sudan, Ethiopia, Tanzania, Madagascar, and Eswatini (formerly known as Swaziland). This vegetable diversity is poorly represented in genebanks and requires urgent conservation action.

The large group of gourds—bitter gourd (*Momordica charantia*), sponge gourd (*Luffa aegyptiaca* and *L. acutangula*), bottle gourd (*Lagenaria siceraria*), wax/ash gourd *(Benincasa hispida*), ivy gourd (*Coccinia grandis*), snake gourd (*Trichosanthes cucumerina*), and spiny gourd (*Momordica dioica*)—requires additional collecting with a major focus on landraces from centers of origin and diversity, many of which are threatened by the introduction of hybrid cultivars by seed companies, a process which recently started in many countries in South and Southeast Asia. Collecting led by national genebanks should focus primarily on Bangladesh, Myanmar, Vietnam, and India, countries rich in landrace diversity of gourds and other underutilized traditional crops. Such efforts could be undertaken in the context of FAO’s Regional Initiative on the Zero Hunger Challenge for Asia and the Pacific, which is targeting the hidden treasures embodied in neglected and underutilized species (NUS), calling them Future Smart Food [65]. These foods are considered smart as they can bolster dietary diversification, improve micronutrient intake, require fewer inputs such as chemical fertilizers, enhance soil health, and often provide resilience to climate change and adverse farming conditions. Based on national NUS scoping studies, eight countries in South and Southeast Asia already prioritized up to six promising NUS as candidates for future smart food.

### 3.2. Linking Genebanks with the Farming Communities

To combat malnutrition, there is a clear need for supportive policies to advance ex situ and on-farm/in situ conservation and documentation of underutilized traditional crops and to forge stronger links between genebanks and the farming communities to strengthen the production and consumption of a wide range of diverse vegetables and fruit trees. In formal seed systems, genebanks usually supply genetic diversity primarily to plant breeders and research organizations who act as intermediaries between them and the farming communities based on a multi-step system of selection, breeding, testing, marketing, and adoption [66]. Today, genebanks are serving an expanded range of actors and institutions, beyond the conventional R&D set-up as outlined in the revised international Genebank Standards published in 2014: “Genebanks should promote the availability of genetic resources for uses including research, breeding, education, farming, and repatriation” [67] (p. 54). Research undertaken by Westengen et al. [68] showed that farmers, farmer organizations, and non-governmental organizations (NGOs) comprise a considerable user group of genebank material, having received about the same percentage (8%) of seed samples distributed by international genebanks in 2015 as distributed to the commercial seed sector. They identified six potential direct genebank–farmer linkages [68]: (1) reintroduction, (2) emergency seed interventions, (3) community seed banks, (4) participatory plant breeding, (5) variety introduction, and (6) integrative seed system approaches. In addition to the common role of genebanks to provide diverse crop germplasm to breeders and, thus, feeding the formal seed sector, such alternative and complementary strategies would strengthen the informal seed sector by enhancing farmers’ access to crop diversity. Two of these genebank–farmer linkages, i.e., community seed banks and variety introduction, are described below.

#### 3.2.1. Community Seedbanks for Locally Important Crop Diversity

Community seedbanks (CSB) can be described as structures in which organized groups of farmers are responsible for the different stages of the management of seed or vegetative propagules, such as selection, conservation, multiplication, exchange, and improvement [69]. CSBs were successfully established all over the world in the last few decades, often supported and funded by NGOs. The main functions of CSBs include (a) conserving and reintroducing predominantly local germplasm, (b) providing easy access to seeds for members of the community, and (c) enhancing seed and food sovereignty [70]. If responsibly managed and supported, such CSBs can support the local community effectively and serve as a reliable seed source of locally important germplasm. Even in Europe, community seed banks are being established at a rapid pace, with at least 130 initiatives reported in 2017 [71].

Key elements in ensuring sustainability for community seedbanks include capacity development to ensure management quality, sustainable mechanisms, i.e., voluntary (in-kind) contributions by farmers to reduce dependence on external funding, enabling legal and political framework conditions to ensure a safe legal basis for operation, enabling social structures among those involved, satisfactory physical infrastructure, and systematic planning processes with effective operational mechanisms.

Community seedbanks and on-farm conservation efforts of crop diversity are only successful if they are fully embedded within the farmers’ livelihood strategies and support the production of nutritious food, the generation of income, and other benefits such as ecosystem services, and socio-cultural and religious practices. Successful examples were documented in a community-based agrobiodiversity management project across Latin America and Southeast Asia [24].

A project on community-based seed production of traditional vegetables in the Philippines provided technical support to farmers for the conservation and multiplication of traditional vegetables to ensure the availability of high-quality seed of promising lines for home and school gardens and commercial production [72]. The project introduced several varieties of five traditional vegetable crops from the WorldVeg genebank: jute mallow (*Corchorus olitorius*; six varieties); cucumber (*Cucumis sativus*; two varieties); bottle gourd (*Lagenaria siceraria*; four varieties); eggplant (*Solanum melongena*; six varieties); ridged gourd (*Luffa acutangula*; three varieties). These crops were complemented with farmer-saved seed of other popular local traditional vegetable crops, such as Lima bean (*Phaseolus lunatus*), butterfly pea (*Clitoria ternatea*), and vegetable hummingbird (*Sesbania grandiflora*). The project made a significant contribution to halting the on-going threat of genetic erosion of local landraces and semi-wild vegetable crops. It also empowered farmers, especially women, to save, use, exchange, and sell their seeds to sustain the diversity of crops grown on-farm and promote greater diversity in diets.

To create the necessary legal foundation for CSBs, it is recommended that national governments integrate farmers’ rights to save, use, exchange, and sell farmer-saved seeds in national seed legislations [73]. In this way, CSBs represent a form to implement farmers’ rights. CSBs also play an intermediary role between genebanks and local seed systems as they multiply the relatively small seed samples usually distributed by genebanks to seed lots that are big enough for the needs of farmers [68].

#### 3.2.2. Variety Introduction of Agricultural Crops in General and Vegetable Crops in Particular

Variety introduction efforts are sometimes triggered by the need to provide farmers with a range of crop species and varieties to help them cope with environmental limitations in a changing climate by matching varieties to diverse production conditions and weather extremes [74]. To strengthen food security in sub-Saharan Africa (SSA), van Etten [74] proposed a crowd-sourcing approach to seed-based innovation, starting with the distribution of many small seed packets of different crops and varieties consisting of farmer varieties, landraces, and modern cultivars. For their distribution, existing networks such as retail stores, NGOs, and churches could be used. After having grown out the seed samples, farmers should provide basic agronomic data on the different varieties using their mobile phones. Farmers could also be requested to provide feedback on the performance of their own varieties compared to the newly introduced varieties. If local varieties prove to be superior to the introduced varieties, they could, if farmers agree, be included in the next evaluation round. This would then lead to a constant exchange of diverse varieties among the farming communities.

Such an approach was initiated in 2009 by Bioversity International through a project called Seeds for Needs (S4N) with project sites in 13 countries in Africa, Asia, and Latin America [68]. The project used crowdsourcing to evaluate both landraces and advanced cultivars and showed that landraces have great potential by offering valuable options to farmers having to deal with climate-related risks [75].

In Spain, the National Center for Plant Genetic Resources (CRF) of the National Institute for Agricultural and Food Research and Technology (INIA) recently launched an initiative to engage farmers, farmer associations, and relevant companies in the primary evaluation of germplasm that is conserved by the national ex situ collection network [76]. This initiative is linked to the First Action Plan of the National Program for the Sustainable Conservation and Use of Genetic Resources for Food and Agriculture (Order APA/63/2019). CRF will make selected varieties of local crop germplasm available to the farming community for cultivation under diverse growing conditions. In return, farmers will share information on the performance of the varieties with CRF, providing data on yield, incidence of diseases, insect pests and weeds, organoleptic quality, and other relevant data. This initiative is expected to strengthen on-farm conservation of valuable crop germplasm and to enhance agrobiodiversity, in line with SDG 2 (Zero Hunger).

The World Vegetable Center came to similar findings with variety introduction in Central Asia and the Caucasus (CAC). Starting from 2005, national research institutes in the CAC region received over several years a total of 2103 breeding lines and landraces of different vegetable crops from the World Vegetable Center [77]. About 45% of materials sent by WorldVeg were genebank accessions, mostly landraces and farmers’ varieties [78]. The institutes undertook selection and adaptation trials with the materials received, and these efforts led to the registration and official release of 91 vegetable varieties as of 2017. Of these 91 new cultivars, 32 (35%) were developed from WorldVeg genebank accessions, 57 came from breeding lines, and only two resulted from actual crosses made using WorldVeg breeding lines. Meanwhile, another 10 varieties are already submitted for registration and subsequent release. The success of research institutes in releasing genebank accessions as varieties, without further cross-breeding, can be attributed to at least four factors [77]: (1) a relatively large number of accessions were tested for their performance under local conditions; (2) extensive selection trials were conducted to purify lines and stabilize traits by selecting for disease resistance and yield performance for at least three years; (3) disease pressure is relatively low in the CAC region due to favorable local climatic conditions (cold winters, hot and dry summers); (4) none of the institutes had well-functioning breeding pipelines at the start of this variety introduction process. Selection trials were, therefore, an effective way to create new cultivars.

The WorldVeg genebank of predominantly traditional vegetables in Arusha, Tanzania used a similar approach of variety introduction into East Africa via vegetable seed kit distribution. Between 2013 and 2017, this genebank distributed more than 42,500 seed kits totaling 183,193 seed samples to smallholder farmers in Tanzania, Kenya, and Uganda [79]. The seed kits usually comprised about four seed samples of different vegetable crops/varieties, enough to grow out in a home garden. About 32% of the seed distributed came from promising accessions of the genebank, while 68% were WorldVeg breeding lines. All materials distributed, including breeding lines, were maintained by the genebank. The WorldVeg genebank in Arusha used five important criteria when composing the seed kits [80]:

(1) Only those accessions and breeding lines were selected for the vegetable seed kits which had undergone screening for yield, disease resistance, and consumer preference under local conditions in Tanzania.
(2) Distributed accessions and breeding lines were open-pollinated so that farmers could save seed for the next cropping cycle or for sharing extra seed with other famers in the community.
(3) Seed distribution channels were international NGOs, farmer groups, and local government units and WorldVeg development projects. Seed kit distribution was not intended as emergency seed aid. The distribution through projects was related to home garden projects intended for improving dietary diversity and diversifying incomes.
(4) Seed kits were distributed together with capacity building in vegetable production and seed saving provided by NGOs or project staff. Seed kits also contained instructions for good agricultural practices for the successful cultivation of the crops and information on the nutritional value of the crops included in the seed kits.
(5) Seed kits were distributed only once to the same household. A regular supply of seed was not envisaged to avoid damaging local seed enterprises which later picked up seed production and sale of some traditional vegetables.

There is a clear trend in some East African countries (Kenya, Tanzania, Uganda) that seed companies become interested in multiplying and selling traditional African vegetable crops due to high demand from the farming community and consumers. To enhance vegetable diversity grown by smallholder farmers across SSA, the WorldVeg genebank in Arusha strongly engages with partners of the formal, as well as the informal, seed sectors.

## 4. Vegetable Genetic Resources as Building Blocks for Vegetable Breeding

Successful breeding, in general, requires access to a wide diversity of plant genetic resources, predominantly farmers’ varieties, landraces, and crop wild relatives (CWR), the building blocks of intra- and interspecific crop diversity. Such plant genetic resources represent a treasure trove of genes for vegetable and legume crop improvement [80], enabling the delivery of more nutritious quality food in sufficient quantity for the world population.

Vegetable breeding should address the needs of both growers and consumers. Vegetable growers appreciate cultivars with high yield, uniformity, good market acceptance, multiple disease and pest resistance, and abiotic stress tolerance. Consumers like to buy vegetables with good appearance, shelf life, quality, taste, and nutritional value. Increased phytonutrient density in vegetable crops could help overcome micronutrient malnutrition and improve human health. The tomato breeding program at the World Vegetable Center includes enhanced phytonutrient content as a breeding objective, and it developed high-yielding and multiple disease-resistant lines with increased content of beta-carotene, lycopene, flavonoids, or anthocyanin in different fruit types [81]. WorldVeg breeders used the *Beta* allele from the wild species *Solanum hirsutum* which shifts tomato carotenoid from lycopene almost entirely to beta-carotene and results in orange fruit color. So-called “golden tomatoes” developed by WorldVeg breeders through conventional breeding techniques contain 3–6 times more provitamin A carotenoids than standard tomatoes, and one golden fresh market tomato can provide a person’s full daily vitamin A requirements. WorldVeg *Beta* breeding lines in fresh market and cherry tomato fruit types were officially released as cultivars in Mali, Taiwan, and Bangladesh [81]. However, adoption is low so far as consumers are unaware or reluctant to accept the unfamiliar orange fruit color. Many major genes affecting nutrient content in tomato are known, and there is significant scope to enhance phytonutrient content in this crop through conventional breeding [81], but few breeding programs pursue better nutrition in their breeding objectives due to lack of market incentives and not strongly enough articulated consumer demands.

Sustainable intensification of horticultural crops requires the development of new varieties with stable yields under climate change scenarios and adaptive capacity to diverse agro-ecosystems. The narrow genetic base of many vegetable crop cultivars is a major challenge for breeders aiming to develop improved varieties with multiple disease and insect pest resistance and tolerance to abiotic stresses such as heat, salinity, and drought, as well as increased input-use efficiency. Those breeding objectives require making use of interspecific crop diversity. Zhang et al. [82] were able to develop interspecific bridge lines among *Cucurbita pepo*, *C. moschata*, and *C. maxima*. With the development of these lines, it was possible to overcome the crossing barriers of interspecific hybridization and to eliminate the sexual obstacles of subsequent generations. This important breakthrough created a platform for breeders to transfer favorable traits among these species freely without the introgression of undesired characters from a wild species during the breeding process.

In contrast to public breeding programs such as those implemented by WorldVeg, for example, private seed companies primarily focus on the development of hybrid cultivars by exploiting heterosis effects and building multiple biotic stress resistance factors, as well as tolerance against abiotic stresses into a single commercial cultivar. This process ensures that growers must purchase fresh seeds for each growing cycle and the control of the parents prevents other seed companies from reproducing the hybrids. At the global level, the share of hybrid seed production is growing at a fast pace of 8–10% per annum in most major vegetable crops [83]. The global vegetable seed market was valued at US$ 9.163 billion in 2018 and is projected to increase at a compound annual growth rate of 9.4% during the period from 2019 to 2024 [84]. North America is among the largest markets for vegetable seed production and consumption, followed by the Asia-Pacific region and Europe. Tomato, cabbage, sweet pepper, and lettuce are key players in the global seed market with a share of more than 30%.

Often, private seed companies make use of public breeding products when developing hybrid cultivars. A typical case is chili pepper (*Capsicum* spp.) variety development in India. Hybrid cultivars account for about 25% of the total area under chili pepper cultivation, about 25% of the area is under improved open-pollinated varieties (OPVs), and the remaining 50% area is still grown with local landraces [85]. The current chili pepper hybrid seed market in India is estimated at about 50 t per year, with an estimated turnover worth 16 million US$, and this was made possible thanks to the use of WorldVeg cytoplasmic male sterility (CMS) breeding lines which reduce the cost of hybrid seed production by 50% compared to conventional hybrid seed development using manual emasculation. Conservative estimates suggest that hybrids involving WorldVeg germplasm and improved breeding lines as one of the parents were cultivated on more than 30,000 ha during 2012–2013 in different regions of India [85].

The potential of wild species as a source of genetic variation to bring about crop improvement was recognized early in the 20th century but is not yet widely used in crop breeding. An exception is perhaps the model crop tomato, which, in terms of production volume, is the most important vegetable crop grown worldwide. For this crop, an enormous amount of biotic and abiotic stress tolerance traits were already studied in the pool of wild relatives and extensively used in tomato breeding. Virtually all significant resistance genes to tomato diseases were sourced from wild relatives. An overview of such disease resistance genes introduced from wild species into cultivated tomato was provided by Ebert and Schafleitner [86]. However, it is essential to strengthen similar research in other major and minor vegetable crops as well.

Initially, molecular breeding complemented conventional breeding methods through marker-assisted selection (MAS) or marker-assisted backcrossing (MABC) [83]. Molecular markers intricately linked to the trait of interest can be detected and used in gene pyramiding, thus facilitating introgression of desirable, mostly monogenic traits from exotic germplasm into elite cultivars. Relatively little work was done with respect to traits that are governed by quantitative trait loci (QTLs). Traits such as yield, quality, and stress response show complex inheritance patterns that result from the segregation of numerous interacting QTLs, the expression of which is modified by the environment [87].

New breeding approaches such as “introgressiomics” allow the creation of highly diverse plant materials and populations carrying introgressions of genome segments from mostly wild crop relatives into the genetic background of crops [88]. Introgressiomics can lead to the development of chromosome substitution lines (CLs), introgression lines (ILs), and multi-parent advanced inter-cross (MAGIC) populations. Such materials can be directly used in breeding pipelines and will facilitate the development of new resilient cultivars.

ILs contain the full genome of a given crop, except for a small chromosomal segment of a wild donor parent [87]. ILs are obtained through repeated backcrossing of the hybrid to the recurrent parent. Molecular markers help tracking the introgressed fragments, thus supporting the selection of beneficial materials for subsequent backcross cycles. A final step in the development of ILs is selfing or obtaining doubled haploids to fix the introgressed fragment in a homozygous state [89]. A further advantage of ILs is the ability to intercross favorable traits that are present in different ILs for the pyramiding of desirable alleles such as yield QTLs in tomato [90].

As early as 1995, Eshed and Zamir [91] developed a novel tomato population consisting of 50 ILs originating from a cross between the drought-tolerant, green-fruited wild tomato species *Lycopersicon pennellii*, and the elite tomato inbred line M82. Each of the lines contains a single homozygous restriction fragment length polymorphism-defined *L. pennellii* chromosome segment and, together, the lines provide complete coverage of the genome and a set of lines nearly isogenic to M82. These nearly isogenic lines of the IL population provide increased sensitivity for QTL mapping compared to whole-genome segregating populations, and they were extensively used during the last two decades to map QTLs for diverse tomato traits [92].

Only recently, the first eggplant introgression line population was developed using the drought-tolerant wild species *S. incanum* as a donor parent [93]. Sixty-eight candidate genes involved in drought tolerance were identified in the set of 25 fixed ILs. Apart from drought tolerance, *S. incanum* is also known to have a high content of bioactive phenolic compounds [94], as well as resistance to some diseases [95]. Open-field and screenhouse evaluations of the eggplant IL population mentioned above revealed that desirable traits such as lack of prickles and yield did not undergo considerable changes in most ILs despite the introgression of relatively large fragments from the wild exotic parent [96]. Ten stable QTLs distributed across seven chromosomes were detected, and three of the fruit-related QTLs appeared to be syntenic to other ones previously reported in eggplant populations. The other seven stable QTLs are new ones demonstrating that eggplant ILs are highly relevant for eggplant breeding under different environments and climatic conditions.

Genomics-assisted breeding is increasingly facilitating the introgression of favorable genes and QTLs with complex inheritance patterns from wild species into cultigens. The long-term conservation of genetic resources of landraces and crop wild relatives, their full characterization and evaluation, and their availability and accessibility will be instrumental for their successful use in public and private breeding programs. Such mobilization of the biodiversity available in the wider crop genepools will allow breeders to develop varieties that bear multiple disease and insect resistance and are able to adapt to rapidly changing environmental conditions, thus boosting agricultural production and ensuring food and nutrition security.

Moving such germplasm, once identified, across borders also requires robust phytosanitary capacity and practices and appropriate distribution capacities of genebanks, especially in the case of crops with recalcitrant seeds or those that are predominantly vegetatively propagated. Effective use of crop wild relatives and landraces also requires strong research capacity and participatory pre-breeding approaches. To enhance adoption, farmers should be actively involved in the definition of breeding objectives and the selection process for the development of new varieties, either through conventional or molecular breeding methods.

In contrast to the common vegetables grown globally, traditional vegetables, especially those which originated in Africa and the Asia-Pacific region receive much less attention from the research, conservation, and breeding community, although they have the potential to play a much greater role in more nutrition-oriented agriculture [97]. Neglect by research and breeders applies to many underutilized fruit and vegetable species. These include those crops with perennial growth forms such as trees, e.g., drumstick tree (*Moringa oleifera* [55]), and shrubs with edible leaves which are suited for agroforestry systems that may have an increasing role to play in sustainable vegetable production systems in developing countries under climate change scenarios.

Compared to major staple food crops, relatively little investment was made in breeding traditional, underutilized crop varieties [98]. Hence, the latter typically do not meet modern standards for uniformity and other characteristics required in the marketplace, and they tend to be less competitive than globally grown and traded crop cultivars. Farmers’ varieties, landraces, and CWR were hitherto increasingly valued and exploited for genes that provide increased biotic resistance, tolerance to abiotic stress, yield, and quality [99,100,101]. However, use of agricultural biodiversity should not be restricted to exploiting valuable genes for use in breeding programs if our aim is to create more robust and resilient production systems. Underutilized food sources, including fruit and vegetables, legume crops, and root and tuber crops, have the potential to make a substantial contribution to food and nutrition security, protect against internal and external market disruptions and climate uncertainties, and lead to better ecosystem functions and services, thus enhancing crop and farming sustainability [102].

## 5. Conclusions

Vegetable crops are key sources of essential micronutrients required for good health. They contribute variety, flavor, taste, and nutritional quality to human diets. Increasing production and consumption of vegetables constitutes a direct and affordable way to deliver better health and overcome malnutrition. Vegetable production has the potential to generate more income and employment than any other segment of the agricultural economy. Vegetables can be grown on small areas of land, close to the consumers in urban and peri-urban settings, and they do not necessarily need advanced technologies to grow them. To realize those benefits, governments and donors need to give more weight and support to the ex situ, on-farm, and in situ conservation of genetic resources (farmers’ varieties, landraces, and CWR) of global, as well as traditional, vegetables. The effective utilization of those genetic resources in breeding programs and the testing and deployment of newly developed varieties with tolerance to abiotic stresses and resistance against multiple diseases and insect pests in farmers’ fields will ultimately benefit the farming community and consumers. By doing so, a significant reduction or, even better, a complete elimination of the obvious and persistent gap between WHO-recommended and actual intake levels of (fruit and) vegetables would make a significant contribution to the achievement of Sustainable Development Goals related to food and nutrition security and good health, in particular SDG 2.3 aiming at doubling agricultural productivity and incomes of small-scale food producers, SDG 2.4 ensuring sustainable food systems, and SDG 2.5 maintaining genetic diversity.
